# Genome-wide analysis of valine-glutamine motif-containing proteins related to abiotic stress response in cucumber (*Cucumis sativus* L.)

**DOI:** 10.1186/s12870-021-03242-9

**Published:** 2021-10-25

**Authors:** Nan Shan, Zijin Xiang, Jingyu Sun, Qianglong Zhu, Yao Xiao, Putao Wang, Xin Chen, Qinghong Zhou, Zengyu Gan

**Affiliations:** 1 Agronomy College, Jiangxi Agricultural University, Nanchang, 330045 China; 2 Jiangxi Key Laboratory for Postharvest Technology and Nondestructive Testing of Fruits and Vegetables, Collaborative Innovation Center of Postharvest Key Technology and Quality Safety of Fruits and Vegetables, Jiangxi Agricultural University, Nanchang, 330045 China

**Keywords:** VQ motif, CsVQ proteins, Plant growth, Abiotic stress

## Abstract

**Background:**

Cucumber (*Cucumis sativus* L.) is one of the most important economic crops and is susceptible to various abiotic stresses. The valine-glutamine (VQ) motif-containing proteins are plant-specific proteins with a conserved “FxxhVQxhTG” amino acid sequence that regulates plant growth and development. However, little is known about the function of VQ proteins in cucumber.

**Results:**

In this study, a total of 32 CsVQ proteins from cucumber were confirmed and characterized using comprehensive genome-wide analysis, and they all contain a conserved motif with 10 variations. Phylogenetic tree analysis revealed that these CsVQ proteins were classified into nine groups by comparing the CsVQ proteins with those of *Arabidopsis thaliana*, melon and rice. *CsVQ* genes were distributed on seven chromosomes. Most of these genes were predicted to be localized in the nucleus. In addition, *cis*-elements in response to different stresses and hormones were observed in the promoters of the *CsVQ* genes. A network of CsVQ proteins interacting with WRKY transcription factors (CsWRKYs) was proposed. Moreover, the transcripts of *CsVQ* gene were spatio-temporal specific and were induced by abiotic adversities. *CsVQ4*, *CsVQ6*, *CsVQ16–2*, *CsVQ19, CsVQ24*, *CsVQ30*, *CsVQ32*, *CsVQ33*, and *CsVQ34* were expressed in the range of organs and tissues at higher levels and could respond to multiple hormones and different stresses, indicating that these genes were involved in the response to stimuli.

**Conclusions:**

Together, our results reveal novel *VQ* resistance gene resources, and provide critical information on *CsVQ* genes and their encoded proteins, which supplies important genetic basis for VQ resistance breeding of cucumber plants.

**Supplementary Information:**

The online version contains supplementary material available at 10.1186/s12870-021-03242-9.

## Background

Cucumber (*Cucumis sativus* L.) is one of the most important economic crops worldwide and is popular and favored by consumers for its distinct aromas and flavors.

However, cucumber is vulnerable to various abiotic stresses, such as drought, low temperature, and salt during the growth and development process. Plants have gradually developed a complex set of mechanisms to adapt to adversity [1, 2], including the regulation of characteristic genes [3]. Valine-glutamine (VQ)-motif-containing proteins, characterized by a highly conserved core sequence FxxhVQxhTG (h denotes hydrophobic amino acid, x means any amino acid), were reported to play crucial roles in plant growth, development, and responses to stresses [4]. The first VQ protein, AtVQ23 (formerly named SIB1, sigma factor binding protein1), was discovered from Arabidopsis [5]. Subsequently, members of *VQ* gene family have been discovered and characterized in diverse plants, including Arabidopsis (34) [6], rice (40) [7], soybean (74) [8], Chinese cabbage (29) [9], bamboo (61) [10], tomato (26) [11], strawberry (25) [12], tea (25) [13], apple (49) [14], grapevine (18) [15], and melon (30) [16]. Based on the sequence features and homology of the VQ domains, VQ family members were classified into seven [7, 9, 10, 12, 13] or ten [11, 14–18] subfamilies.

Accumulated evidences have demonstrated that VQ proteins serve as critical regulators in multiple biological processes, including development of endosperm and pollen [19–21], seed germination and early seedling establishment [22], photomorphogenesis [23], chloroplast development or photosystem assembly [6], and jasmonic acid (JA) or salicylic acid (SA) -mediated disease resistance [24–27]. In addition to these, lots of *VQ* genes have been evidenced to play versatile functions in response to abiotic stresses [4]. Evidences show that *AtVQ9* and *AtVQ15* negatively regulate salt and osmotic tolerance in Arabidopsis [28, 29]. Similarly, *MaVQ5* gene in banana represses the transactivation of JA biosynthetic genes in response to cold stress [30]. *SlVQ6*-overexpressing plants exhibited a high sensitivity to high temperature stress, indicated that *SlVQ6* gene in tomato negatively regulates thermotolerance [11]. In contrast, *PeVQ28* was found to act as a positive regulator for salt stress and abscisic acid (ABA) signal pathway [31]. *IbVQ4* may participate in the drought and salt tolerance in sweet potato [32]. *ZmVQ* genes are responsive to abiotic stress [33], and *PeVQ* genes are differentially regulated by polyethylene glycol (PEG), ABA, and SA treatment in Moso bamboo [10].

Many VQ proteins participate in stress resistance by interacting with other proteins, especially with WRKY transcription factors [4]. As the largest family of transcriptional regulators in plants, WRKY transcription factors regulate plant biological processes and respond to various biotic and abiotic stresses by interacting with the conserved V and Q residues of the VQ proteins [34]. It was reported that AtVQ10 interacts with WRKY8 to modulate the basal defense against *Botrytis cinerea* in Arabidopsis [35]. Apple MdVQ protein was shown to bind with Group I and IIc MdWRKYs [14]. Another mode of VQ proteins is as a downstream substrate of mitogen-activated protein kinases (MAPKs). Previous studies showed that VQ proteins seem to bridge MAPK and WRKY transcription factors to form a ternary complex (WRKY-VQ-MAPK). AtVQ21/ MKS1 is first identified to be phosphorylated by AtMPK4, and AtVQ21, AtMPK4 and specific WRKY transcription factors (WRKY25 and WRKY33) co-regulate plant resistance through the complex interaction [18]. Tomato SlVQ6 has identified as a substrate of SlMPK1 and responds to abiotic stresses such as drought, high temperature and salt stresses [11]. In addition, other regulatory mechanisms of VQ proteins are increasingly being revealed. AtVQ29 restrains seedling de-etiolation by coacting with phytochrome-interacting factor1 [23]. During seed germination, AtVQ18 and AtVQ26 interact with the transcription factor ABA-insensitive5 to negatively regulate the ABA response in Arabidopsis [22]. The ring-type E3 ubiquitin ligase JUL1 (JAV1-associated ubiquitin ligase1) aims the VQ-motif protein to coordinate JA signaling pathway [36].

Although extensive efforts have been performed to investigate molecular mechanism of VQ protein involved in abiotic stress response in various plants, only a few of these genes have been characterized functionally to date and a large number of the VQ family members have not yet been studied. Particularly, *VQ* genes in cucumber have been reported little. The draft genome sequence of cucumber, assembled using a combination of traditional Sanger and next-generation Illumina GA sequencing technologies, was obtained in 2009 [37], which affords insight into traits such as its stress resistance. However, some drawbacks, including the redundancy of repetitive DNA sequences, were found in the first assembly of the cucumber genome. Based on the RNA-Seq reads, the prediction of protein-coding genes was improved in the reassembled cucumber genome (cucumber ‘Chinese Long’ v2 genome) [38]. In recent years, with the development of new cost-effective and accurate technologies [39], including 10X Genomics, optical mapping, and high-throughput chromosome conformation capture, a chromosome-scale genome assembly of cucumber (cucumber ‘Chinese Long’ v3 genome) was generated [40], which serves as a valuable resource for genetic research in cucumber.

The completion and improvement of cucumber genome sequence provides an excellent opportunity for genome-wide analysis of *VQ* gene family. In the current study, a total of 32 *CsVQ* genes were identified based on cucumber genome (Cucumber ‘Chinese Long’ v2) and transcriptome databases of cucumber. These *CsVQ* genes were clustered into 9 subfamilies based on phylogenetic analysis. Then their conserved motifs, conserved domains, gene structure, functional interaction network, and spatio-temporal expression patterns were comprehensively studied. To identify *CsVQ* candidate genes associated with abiotic stress, transcripts of nine *CsVQ* genes were examined under cold, drought, salinity and hormone treatments. These results indicated that CsVQ proteins not only involved in plant growth and development regulation, but also in abiotic stress and hormone treatment. In summary, this study would provide comprehensive information about *CsVQ* genes from cucumber, as well as the insights for the further functional investigation and application of novel *CsVQ* candidate genes for crop improvement, especially in aspects of stress resistance, growth and development.

## Results

### Gene characterization, phylogenetic tree, and chromosomal locations analysis

**Fig. 1:**
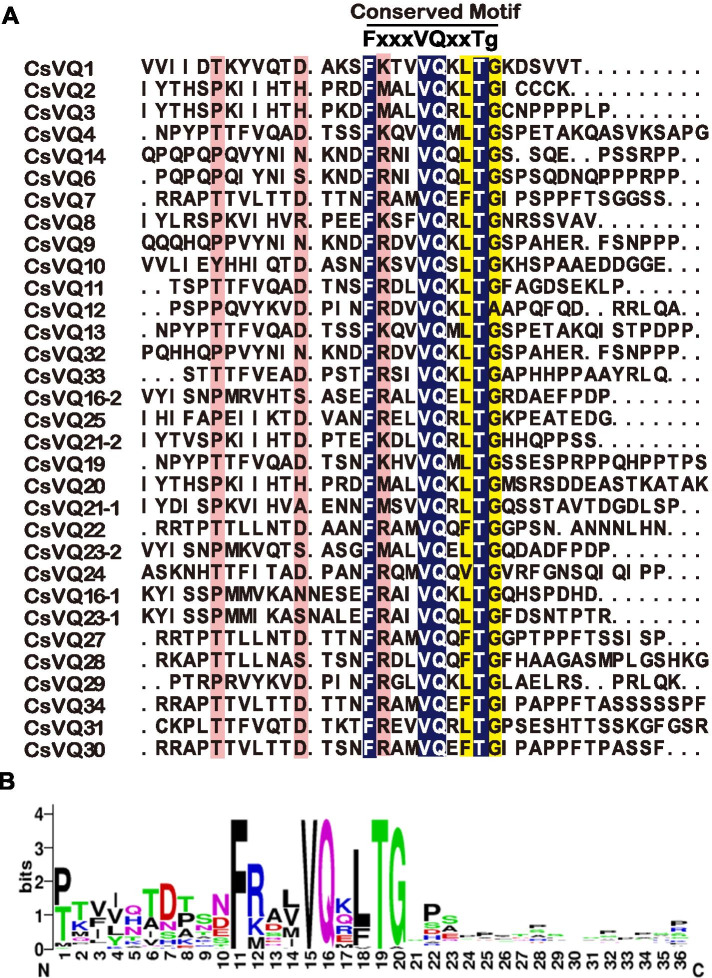
Multiple sequence alignment of CsVQ proteins in cucumber. **A**, sequence alignment of CsVQ proteins using DNAMAN version 6.0, where x represents any amino acid. The conserved amino acid sequence of FxxxVQxLTG is clearly highly conserved and shown in (**B**)

To identify the homologous VQ protein family in cucumber plants, conserved motif (PF05678) of VQ protein was used as a query in Cucumber ‘Chinese Long’ v2 genome of Cucurbit Genomics Database (CuGenDB) [37]. A total of 32 *CsVQ* genes were identified and assigned specific names based on sequence similarity and phylogenetic tree among AtVQ [6], CmVQ [16], SlVQ [11], OsVQ [7], and CsVQ proteins (Fig. 1; Table 1). Protein sequence analysis results showed that all CsVQ proteins shared similar amino acid sequence FxxxVQxL/F/VTG, and four types were identified in cucumber, including FxxxVQxVTG (1/32), FxxxVQxLTA (1/32), FxxxVQxFTG (6/32), and FxxxVQxLTG (24/32) (Fig. 1). Gene locus ID, open reading frame length, and physiological and biochemical properties of 32 CsVQ proteins were analyzed, including length ranging from 81 to 405 amino acids (aa), molecular weight ranging from 9.28 to 42.78 kDa, and theoretical isoelectric point (pI) ranging from 4.37 to 11.80 (Table 1). Furthermore, subcellular localization prediction results showed that most CsVQ proteins were located in the nucleus, whereas a few proteins were located in the mitochondria or cytoplasm (Table 1).

**Table 1:** Properties of the cucumber *VQ* genes and proteins

Gene Name	Gene Locus	ORF length (bp)	Chr. No.	Position	Protein			
					Length (aa)	MW (kDa)	pI	Subcellular location
*CsVQ1*	Csa4G075740	246	4	5,375,816 .. 5,376,158 (+)	81	9.28	9.72	Nucleus
*CsVQ2*	Csa1G023620	645	1	2,381,813 .. 2,382,555 (+)	214	22.99	6.49	Nucleus
*CsVQ3*	Csa5G609750	432	5	23,658,353 .. 23,658,886 (+)	143	15.82	8.76	Nucleus
*CsVQ4*	Csa1G074920	759	1	7,652,045 .. 7,655,640 (+)	252	27.41	10.37	Nucleus
*CsVQ6*	Csa3G011640	969	3	1,171,817 .. 1,173,439 (+)	322	34.98	11.80	Cytoplasm
*CsVQ7*	Csa3G895870	768	3	38,675,299 .. 38,676,094 (−)	255	27.48	8.49	Nucleus
*CsVQ8*	Csa3G785410	339	3	30,519,688 .. 30,520,134 (+)	112	12.33	11.53	Extracell; Mitochondrion
*CsVQ9*	Csa2G369080	1074	2	17,972,789 .. 17,973,921 (−)	357	38.62	10.89	Nucleus
*CsVQ10*	Csa6G523420	375	6	28,092,380 .. 28,092,798 (−)	124	14.08	7.73	Nucleus
*CsVQ11*	Csa4G651800	528	4	22,459,448 .. 22,460,042 (+)	175	19.27	10.96	Nucleus
*CsVQ12*	Csa2G271490	267	2	13,149,552 .. 13,149,818 (+)	88	9.74	10.45	Nucleus
*CsVQ13*	Csa6G517410	675	6	27,253,151 .. 27,253,923 (+)	224	24.34	8.94	Nucleus
*CsVQ14*	Csa3G902300	981	3	38,997,555 .. 38,998,831 (+)	326	35.22	10.52	Extracell
*CsVQ16–1*	Csa2G010160	423	2	1,842,928 .. 1,843,350 (−)	140	15.73	5.90	Cytoplasm; Extracell; Mitochondrion; Nucleus
*CsVQ16–2*	Csa3G820480	435	3	31,922,073 .. 31,922,792 (−)	144	16.41	4.37	Nucleus
*CsVQ19*	Csa4G637130	741	4	20,716,210 .. 20,717,384 (−)	246	36.42	9.08	Nucleus
*CsVQ20*	Csa2G350280	714	2	15,927,811 .. 15,928,547 (+)	237	25.10	6.50	Nucleus
*CsVQ21–1*	Csa2G302100	639	2	14,531,424 .. 14,532,251 (−)	212	22.96	7.84	Nucleus
*CsVQ21–2*	Csa6G338080	534	6	15,427,318 .. 15,427,851 (−)	177	19.37	6.90	Nucleus
*CsVQ22*	Csa4G618460	582	4	19,753,678 .. 19,754,725 (+)	193	20.77	9.13	Nucleus
*CsVQ23–1*	Csa2G010150	420	2	1,836,083 .. 1,836,502 (−)	139	16.05	8.35	Nucleus
*CsVQ23–2*	Csa4G431960	495	4	16,320,651 .. 16,321,291 (+)	164	17.52	4.39	Nucleus
*CsVQ24*	Csa3G816710	723	3	31,696,876 .. 31,697,690 (−)	240	25.98	6.51	Cytoplasm; Extracell; Nucleus
*CsVQ25*	Csa3G807350	1035	3	30,912,350 .. 30,913,425 (−)	344	34.35	4.96	Cytoplasm; Nucleus
*CsVQ27*	Csa4G056730	690	4	4,862,111 .. 4,862,837 (−)	229	24.60	6.35	Extracell; Nucleus
*CsVQ28*	Csa6G526440	564	6	28,778,536 .. 28,779,301 (−)	187	20.15	6.97	Nucleus
*CsVQ29*	Csa6G495810	507	6	24,113,317 .. 24,113,823 (+)	168	18.53	9.57	Nucleus
*CsVQ30*	Csa6G500500	1197	6	24,943,199 .. 24,944,684 (−)	398	42.56	7.28	Nucleus
*CsVQ31*	Csa4G448640	501	4	16,849,697 .. 16,850,197 (+)	166	18.80	10.08	Nucleus
*CsVQ32*	Csa3G889820	1011	3	37,789,436 .. 37,791,027 (−)	336	36.12	10.89	Nucleus
*CsVQ33*	Csa7G432350	423	7	17,187,689 .. 17,188,487 (+)	140	15.09	7.04	Nucleus
*CsVQ34*	Csa4G028990	1218	4	3,005,474 .. 3,007,138 (+)	405	42.78	6.85	Nucleus

**Fig. 2:**
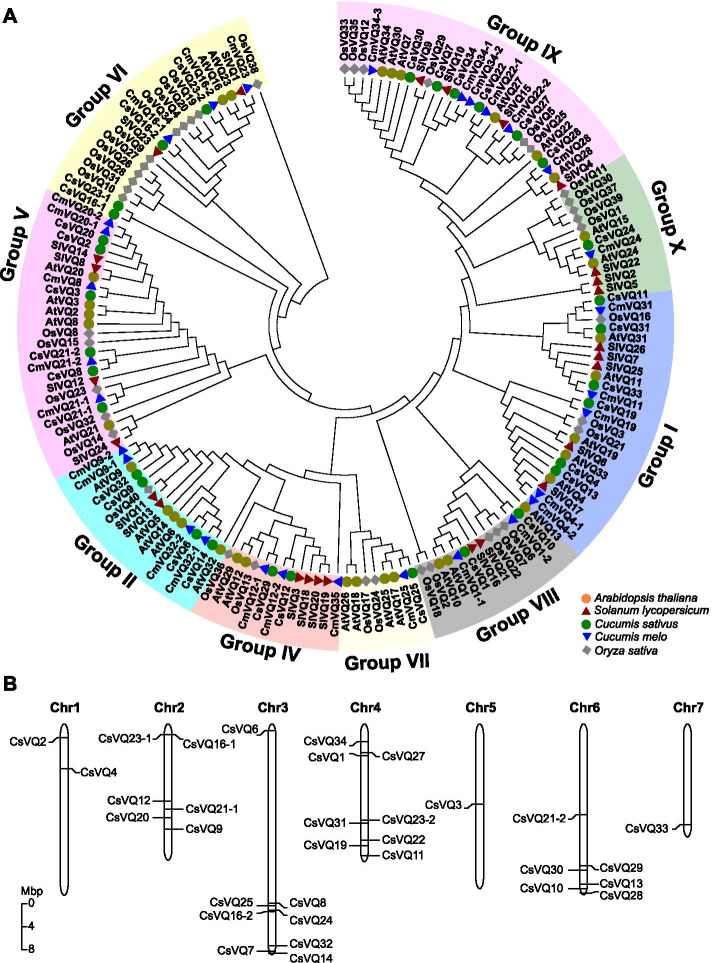
Phylogenetic tree (**A**) and chromosomal locations (**B**) of *CsVQ* genes. **A**, the phylogenetic tree of the 32 cucumber CsVQs, 30 melon CmVQs, 34 Arabidopsis AtVQs, 26 tomato SlVQs, and 40 rice OsVQs was established by using the maximum likelihood method in MEGA X. Proteins from cucumber, melon, Arabidopsis, tomato, and rice are denoted by green dots, blue triangles, brown dots, red triangles, and grey squares, respectively. **B**, the chromosomal locations of the *CsVQ* genes were determined using MapChart 2.3.2

To explore the evolutionary relationship between cucumber CsVQ proteins, a phylogenetic tree was constructed among 32, 30, 34, 40, and 26 VQ proteins of cucumber, melon, Arabidopsis, rice, and tomato, respectively (Fig. 2A). CsVQ proteins of cucumber appeared in 9 groups (I, II, IV–X) according to the classification of Arabidopsis, melon and tomato in the previous studies [11, 16, 18]. Group IX possessed largest number of VQ proteins, including 28 VQ proteins (6 CsVQs, 6 CmVQs, 6 AtVQs, 6 OsVQs, and 4 SlVQs). Group V, the second largest group, consist of 25 VQ proteins (6 CsVQs, 5 CmVQs, 5 AtVQs, 5 OsVQs, and 4 tomato SlVQs). There was only one CsVQ protein in group VII (Fig. 2A). The evolutionary relationship indicates that the CsVQ proteins exhibit a close relationship with the melon and Arabidopsis VQ proteins and a distant relationship with the rice VQ proteins in the same group.

Chromosomal location results showed that 32 *CsVQ* genes were randomly and unequally distributed on 7 chromosomes. Specifically, chromosomes 3 and 4 harbored the largest number of *CsVQ* genes with eight genes, and chromosomes 2 and 6 both possessed six *CsVQ* genes. However, chromosomes 5 and 7 had only one gene each (Fig. 2B). Gene structure analysis results showed that most *CsVQ* genes possessed one exon structure, except that *CsVQ2, CsVQ4, CsVQ7*, *CsVQ9, CsVQ25,* and *CsVQ27* contained two exon structures (Fig. S1).

### Conserved motif analysis of CsVQ proteins

**Fig. 3:**
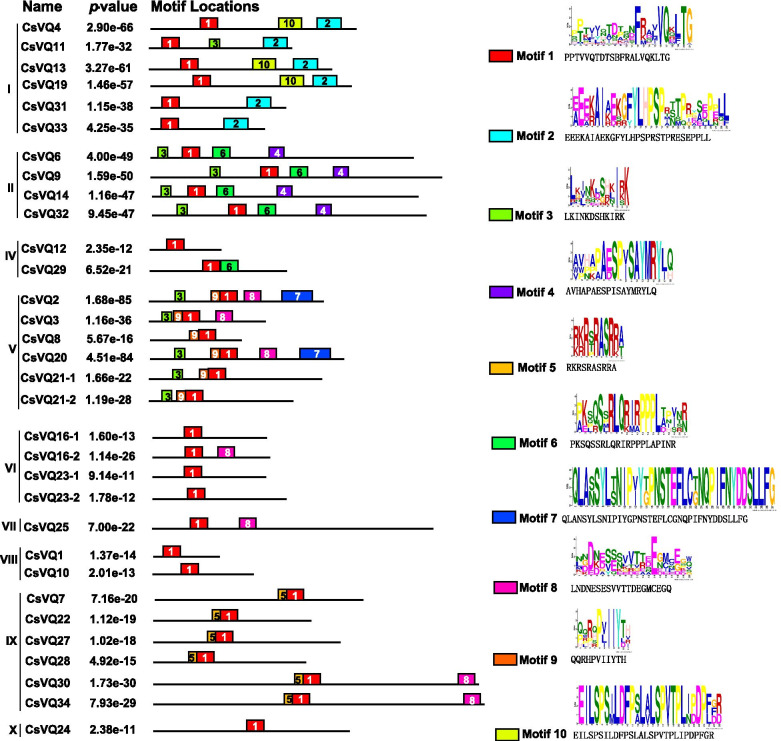
Conserved motif analysis of the CsVQ proteins in cucumber. Distribution of the CsVQ conserved motifs in cucumber was analyzed by MEME with 10 Maximum Number of Motifs (http://meme-suite.org/). Each specific motif is marked by a different colored box, and the names are included in the center of each box

VQ- motif was reported to have a significant impact on the function of VQ proteins, and the site mutation of VQ motif may affect the functions [4]. To investigate the sequence features of CsVQ proteins, conserved motifs were predicted and analyzed using the MEME suit. Ten motifs, ranging from 10 to 38 amino acids, were predicted, and exhibited highly conserved amino acid residues (Fig. 3; Fig. S1). Motif 1 corresponding to the VQ-containing motif was distributed in all CsVQ proteins, and other conserved motifs (motifs 2 to 10) were unequally distributed in CsVQ proteins; e.g., motif 2 in six, motif 3 in ten, motif 4 in four, motif 5 in five, motif 6 in five, motif 7 in two, motif 8 in five, motif 9 in six, and motif 10 in three CsVQ proteins (Fig. 3). Some motifs were present in the proteins of specific groups. For example, motifs 2 and 10 were specifically distributed in CsVQ4, CsVQ13, and CsVQ19 belonging to Group I; motif 4 was specifically distributed in proteins belonging to Group II; motifs 7 and 9 were specifically distributed in CsVQ2, CsVQ3, CsVQ8, CsVQ20, CsVQ21–1, and CsVQ21–2 belonging to Group V; and motif 5 was specifically distributed in proteins belonging to Group IX (Fig. 3). In addition, CsVQ proteins containing similar motifs belonged to the same group, which corresponded with the phylogenetic analysis results (Fig. 2A). Conversely, CsVQ proteins in different groups exhibited significantly different types and numbers of motifs, indicating the structural basis for diversity in protein function.

### Interaction network of CsVQ proteins

**Fig. 4:**
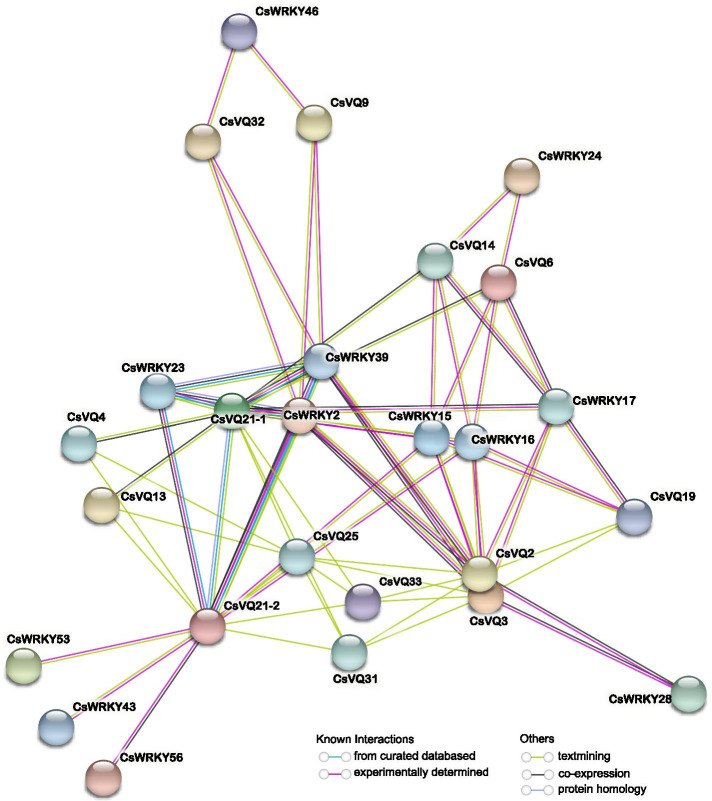
Putative interaction network of CsVQ proteins in cucumber. The prediction of interaction between CsVQ proteins and CsWRKY proteins were integrated using the STRING tool (https://string-db.org/)

**Fig. 5:**
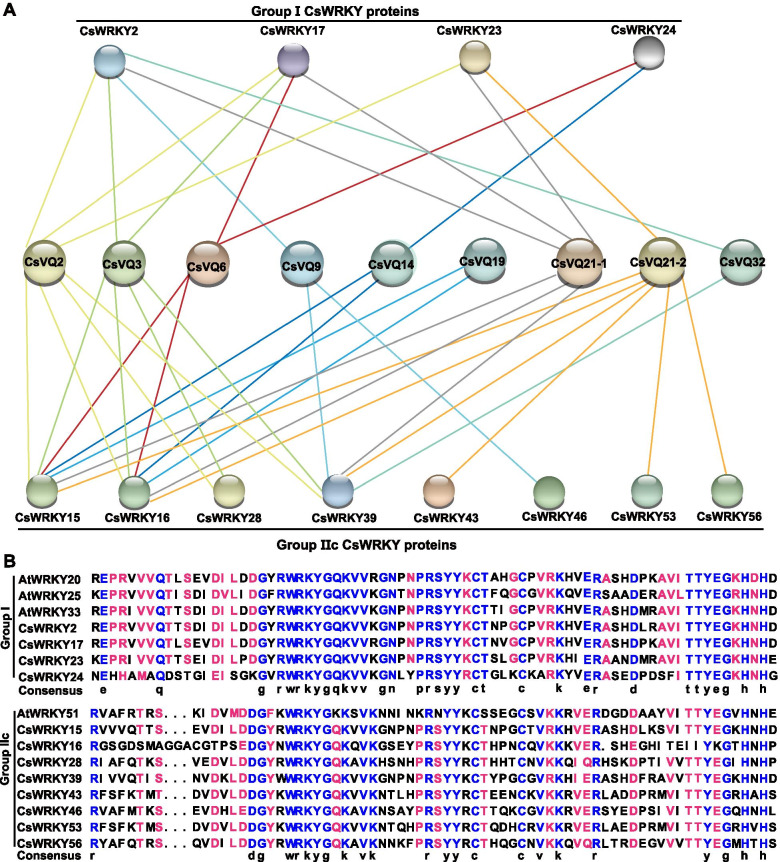
Interaction of CsVQ proteins with CsWRKY transcription factor in cucumber. **A**, the prediction of interaction between CsVQ proteins and CsWRKY proteins was drawn by the PAIR website. **B**, sequence analysis of the C-terminal WRKY domains of group I and group IIc CsWRKY proteins [41]

Interacting with the WRKY family is the common mode of action of VQ proteins. An interaction network of CsVQ proteins was constructed to understand their functional and physical interactions. Fourteen CsVQ proteins and twelve CsWRKY proteins were shown to participate in the interaction network (Fig. 4). To further explore the network, a specific CsVQ-CsWRKY interaction network was constructed (Fig. 5). Nine key nodes, including CsVQ2, CsVQ3, CsVQ6, CsVQ9, CsVQ14, CsVQ19, CsVQ21–1, CsVQ21–2, and CsVQ32, are presumed to interact with different WRKY transcription factors (Fig. 5A). For instance, CsVQ6 was predicted to interact with CsWRKY16, CsWRKY16, and CsWRKY17; CsVQ14 with CsWRKY16 and CsWRKY24; CsVQ21–1 and CsVQ21–2 with CsWRKY2, CsWRKY15, CsWRKY16, CsWRKY17, CsWRKY23, and CsWRKY39. In addition, 12 CsWRKYs were speculated to interact with different CsVQ proteins, including CsWRKY15, CsWRKY16, CsWRKY28, CsWRKY39, CsWRKY43, CsWRKY46, CsWRKY53, and CsWRKY66 belong to group IIc WRKY transcription factor and the others belong to the group I WRKY transcription factor [41] (Fig. 5A). In Arabidopsis, AtWRKY20, AtWRKY25, AtWRKY33, and AtWRKY51 were reported to closely interact with different AtVQ proteins [11]. Multiple sequence alignment of C-terminal WRKY domains of above CsWRKY and AtWRKY proteins showed that the core binding domain of these WRKYs was highly conserved (Fig. 5B), which were considered to be the key to the interaction between VQ and WRKY proteins.

### Expression profiles of *CsVQ* genes in different tissues of cucumber plant

To understand the potential function of *CsVQ* genes, *cis*-elements were predicted. Four types of promoters, including stress-responsive, hormone-responsive, tissue-specific expression, as well as light-responsive promoters, were identified in *CsVQ* genes (Table S1). Stress-responsive elements responded to drought, low temperature, defense and stress, and anaerobic induction (Table S1). Elements were also found to respond to hormones, such as ABA, SA, methyl jasmonate (MeJA), auxin, and gibberellin, indicating that *CsVQ* genes may be regulated by these hormones (Table S1; Fig. S2). Additionally, the promoters of some *CsVQ* genes comprised W-box elements (TTGAC, WRKY-binding sites), suggesting that their transcriptions may be regulated by WRKY proteins (Table S1).

**Fig. 6:**
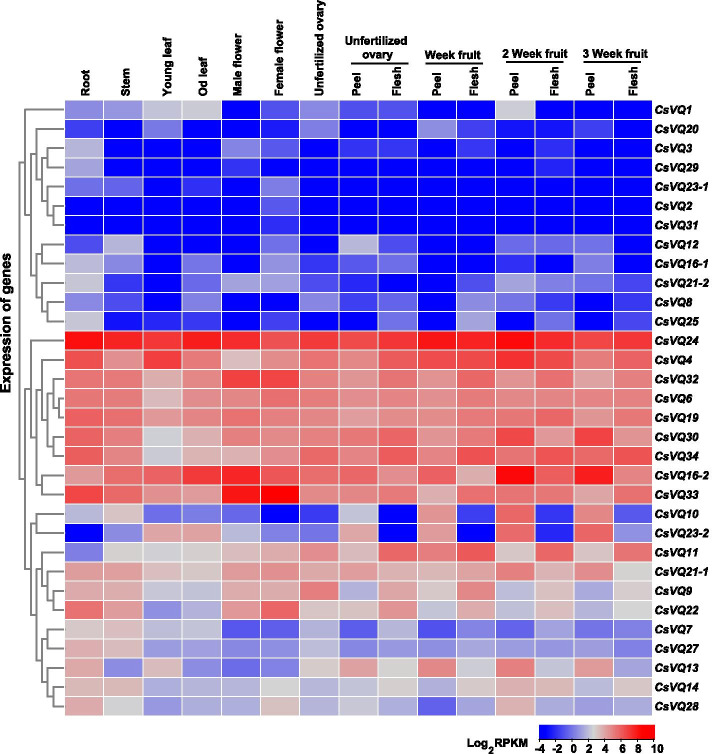
Gene expression profiles in different tissues. Heatmap of 32 *CsVQ* gene expression levels in in different tissues from Cucurbit Genomics Database (http://cucurbitgenomics.org/). The hierarchical cluster dendrogram and heatmap were generated using log2 values of gene expression levels, and color scale for fold-change values was shown. Accession numbers of the gene sequences used for expression analysis are listed in Table 1

To clarify the specificity of tissue expression, the expression levels of each *CsVQ* gene from CuGenDB were examined. The *CsVQ* genes were differentially expressed in the tissues of cucumber plants (Fig. 6). Among 32 *CsVQ* genes, *CsVQ4*, *CsVQ6*, *CsVQ16–2*, *CsVQ19*, *CsVQ24*, *CsVQ30*, *CsVQ32*, *CsVQ33*, and *CsVQ34* were highly expressed, whereas *CsVQ31* and *CsVQ2* had the lowest expression levels (Fig. 6). *CsVQ24* and *CsVQ16–2* exhibited the highest transcription levels in all *CsVQ* genes from selected samples, which was also similar to the *CsVQ* gene expression levels in different tissues of the Chinese long cucumber species (data from bioproject PRJNA80169 of Cucurbit Expression Atlas).

### Expression profiles of *CsVQ* genes in response to hormones

**Fig. 7:**
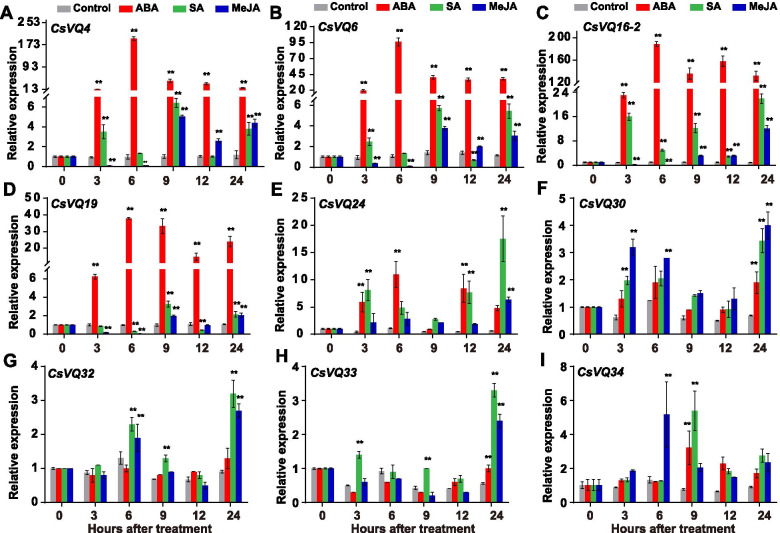
Gene expression analysis of certain *CsVQ* genes under various hormones treatments. Expression of *CsVQ4* (**A**), *CsVQ6* (**B**), *CsVQ16–2* (**C**), *CsVQ19* (**D**), *CsVQ24* (**E**), *CsVQ30* (**F**), *CsVQ32* (**G**), *CsVQ33* (**H**), and *CsVQ34* (**I**) under ABA (red column), SA (green column), and MeJA (blue column) treatments were analyzed respectively. Bars represent means ± standard deviations (*n* = 3); data were analyzed by one-way ANOVA followed by Tukey’s test (** *P* < 0.01, * *P* < 0.05). Accession numbers of the gene sequences used for expression analysis are listed in Table 1, and the primers used for qRT-PCR were shown in Supplementary Table S2

To better understand the potential function of *CsVQ* genes in plant hormones, the responses of *CsVQ4*, *CsVQ6*, *CsVQ16–2*, *CsVQ19*, *CsVQ24*, *CsVQ30*, *CsVQ32*, *CsVQ33*, and *CsVQ34* to SA, ABA, and MeJA treatments were investigated using quantitative real-time polymerase chain reaction (qRT-PCR) (Fig. 7), based on their high expression in roots, stems, male flowers, female flowers, ovaries, and fruits (Fig. 6). The results showed that four *CsVQ* genes, namely *CsVQ4*, *CsVQ6*, *CsVQ16–2*, and *CsVQ19,* were induced by ABA treatment (Fig. 7A-D). Following ABA treatment, *CsVQ4*, *CsVQ6*, *CsVQ16–2*, and *CsVQ19* were significantly upregulated at 3 h post-treatment, reaching a peak at 6 h after treatment, and three of the genes (*CsVQ4*, *CsVQ6*, and *CsVQ19*) maintained an evident downregulation tendency 9 h after ABA treatment (Fig. 7A-D). *CsVQ16–2* expression was strongly promoted by ABA treatment, whereas *CsVQ24*, *CsVQ30*, *CsVQ32*, *CsVQ33*, and *CsVQ34* were barely responsive to ABA treatment (Fig. 7E-I). During SA treatment, *CsVQ16–2* and *CsVQ24* were upregulated at all stages, and *CsVQ4*, *CsVQ6,* and *CsVQ34* (except at 3 h) were significantly upregulated at 3 h, 9 h, and 24 h, whereas *CsVQ19* was slightly downregulated at 6 h and 12 h (Fig. 7). Nevertheless, compared to the before-treatment levels, these nine *CsVQ* genes were highly expressed at 24 h after SA treatment (Fig. 7). Additionally, the expression levels of *CsVQ4*, *CsVQ6*, *CsVQ16–2*, and *CsVQ19* sharply decreased at 3 h and 6 h and significantly increased at 9 h, 12 h, and 24 h after MeJA treatment, and *CsVQ24*, *CsVQ30,* and *CsVQ34* were upregulated at all stages of the MeJA treatment (Fig. 7). *CsVQ4 and CsVQ6* were upregulated 5-fold and 4-fold at 9 h post MeJA treatment, respectively, and *CsVQ16–2* and *CsVQ19* were upregulated 10-fold and 2-fold at 24 h after MeJA treatment (Fig. 7). These results indicate that almost all highly expressed *CsVQ* genes are involved in the cucumber response to hormones; however, it is not known whether they are involved in the adversity response in cucumbers.

### Expression profiles of *CsVQ* genes in response to abiotic treatment

**Fig. 8:**
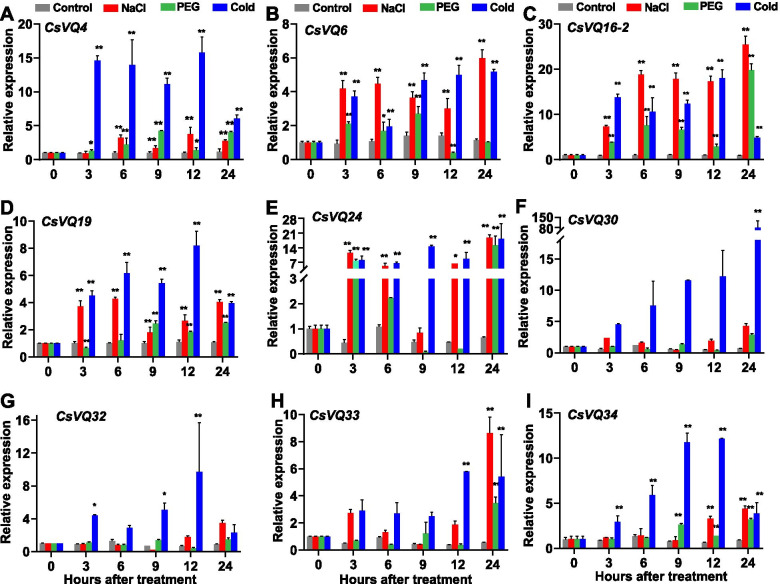
Gene expression analysis of certain *CsVQ* genes under abiotic stress. Expression of *CsVQ4* (**A**), *CsVQ6* (**B**), *CsVQ16–2* (**C**), *CsVQ19* (**D**), *CsVQ24* (**E**), *CsVQ30* (**F**), *CsVQ32* (**G**), *CsVQ33* (**H**), and *CsVQ34* (**I**) under salt (red column), drought (green column), and cold (blue column) treatments were analyzed respectively. Bars represent means ± standard deviations (*n* = 3); data were analyzed by one-way ANOVA followed by Tukey’s test (** *P* < 0.01, * *P* < 0.05). Accession numbers of the gene sequences used for expression analysis are listed in Table 1, and the primers used for qRT-PCR were shown in Supplementary Table S2

To characterize the biological functions of the *CsVQ* genes under salt, drought, and low-temperature stress conditions, the expression levels of *CsVQ4*, *CsVQ6*, *CsVQ16–2*, *CsVQ19*, *CsVQ24*, *CsVQ30*, *CsVQ32*, *CsVQ33*, and *CsVQ34* were detected (Fig. 8). When subjected to salt stress, the expression of *CsVQ4*, *CsVQ6*, *CsVQ16–2*, *CsVQ19,* and *CsVQ24* was significantly upregulated compared to that of the control, although *CsVQ4* transcripts decreased at 3 h, and *CsVQ24* transcripts decreased at 9 h (Fig. 8A-E). Compared with the control, all selected *CsVQ* genes were highly expressed at 24 h after salt stress (Fig. 8). The expression of *CsVQ4*, *CsVQ6*, *CsVQ16–2*, *CsVQ19*, and *CsVQ24* were upregulated after exposure to drought and cold stresses, although the expression of certain genes started declining at 12 and 24 h (Fig. 8A-E). The expression levels of *CsVQ16–2* significantly increased under the three stresses, which were upregulated by 35 times at 24 h after salt treatment, 30 times at 24 h after drought treatment, and 25 times at 12 h after cold treatment, indicating that *Cs16–2* is involved in the response to different environmental stresses, such as salt, drought, and low temperature and may play diverse roles in the adversity response in cucumbers.

Among the three abiotic treatments, the response of *CsVQ4* to cold stress was the most noticeable relative to that to drought and salt stresses, with a 15-fold increase at 12 h after cold treatment (Fig. 8). Moreover, *CsVQ6* and *CsVQ19* were more sensitive to salt and cold stresses than to the drought stress, whereas *CsVQ16–2* responded drastically to all three stresses (Fig. 8). These results suggest that almost all selected *CsVQ* genes are involved in the salt and drought responses of cucumber plants, and the response mechanisms are complex and diverse.

## Discussion

Cucumber is one of the most widely cultivated vegetables in the world, its growth and development were significantly affected by genetic and environmental factors [42]. VQ protein is a plant-specific transcription factor and is involved in plant growth, development, and responses to environmental adversities. In previous studies, the *VQ* gene family has been identified and characterized in Arabidopsis [6], tomato [11], rice [7], soybean [8], Chinese cabbage [9], bamboo [10], strawberry [12], tea [13], apple [14], grapevine [15] and melon [16]. However, detailed information concerning *CsVQ* characters and functions, particularly their role in stresses responses of cucumber, remained unclear. In the present study, 32 *CsVQ* genes were identified in CuGenDB, and the structure and characteristics of *CsVQ* genes and proteins were analyzed. The VQ protein is characterized by the highly conserved amino acid sequence FxxxVQxL/F/VTG, with differences in the regions of leucine (L) and glycine (G). In previous studies, six motifs were identified in Arabidopsis [6], five in soybean [43], six in Chinese cabbage [9], six in maize [33], five in Moso bamboo [10], five in strawberry [44], two in tea plant [13], four in melon [16], and seven in tomato [11]. Sequence analysis of CsVQ protein showed that all *CsVQ* proteins shared a similar VQ domain with four variations (LTG, FTG, VTG, LTA) (Fig. 1), which was the same as that of melon [16]. Furthermore, the VQ domain can affect protein–protein interactions [45], and the mutation of the VQ amino acid residue may invalidate the interaction between VQ and transcription factors [20, 25, 34].

The Arabidopsis VQ protein has been classified into 10 groups based on the evolutionary analysis results [6]. Based on the phylogenetic tree analysis, CsVQ proteins can be divided into nine groups, and there were no members in group III, as in melon [16]. The conserved motifs provided further support for the classification of *CsVQ* genes in cucumber (Fig. 3). The most closely related CsVQ members in the same group showed similarities in motif distribution (Fig. 3). Ten conserved motifs were discovered in all CsVQ proteins, and the conserved motifs were significantly different among different groups of CsVQ proteins. Most CsVQ proteins of the same group apparently had similar motifs constituents (Fig. 3), suggesting that the similarities in conserved motifs in the same subfamily corroborate their classification and inferred evolutionary relationships of these CsVQ proteins. The evolution of the *VQ* gene family is relatively conservative, and a majority of *VQ* genes lose introns during evolution [10], such as in Arabidopsis [6], Chinese cabbage [9], tomato [11], melon [16], and rice [7]. Similar to these findings, most *CsVQ* genes (81.25%) possessed one exon structure, except that *CsVQ2*, *CsVQ4*, *CsVQ7*, *CsVQ9*, *CsVQ25*, and *CsVQ27* contained two exon structures (Fig. S1). In addition, most of *VQ* genes encode relatively small proteins with amino acids less than 300 amino acids, with the exception of *OsVQ34* (1138 aa in Rice), *MdVQ13*, and *MdVQ48* (1076 aa, 2064 aa in Moso bamboo) [10, 17]. In cucumber, almost all CsVQ proteins contain less than 400 amino acids (Table 1). Furthermore, most of VQ proteins are predicted to be located in the nucleus and a few are positioned on the cytoplasm, chloroplast and mitochondria [6, 13, 17]. These researches indicate that VQ proteins in different plants own similarities.

*VQ* genes have been demonstrated to participate in various aspects of growth, development and response to abiotic stresses [4]. *Cis*-acting stress-responsive elements in the promoter region, such as ABRE, W-box, and TCA-element, can well reflect these aspects [46]. Previous and our present studies showed that *cis*-elements exist in response to stress, hormones, and light, indicating the regulation of *VQ* genes to plant growth, stress, hormones, or light [10, 15, 16]. In addition, there is growing evidence that plant VQ proteins achieve biological functions by interacting with proteins such as WRKY transcription factors, one of the largest transcriptional regulatory families that regulate plant growth and development [6, 15, 19, 21, 30, 34, 35, 47]. In Arabidopsis, AtVQ16, AtVQ23 act as activator of AtWRKY33 to positively regulate the plant defense [34], and AtVQ9 forms a complex with AtWRKY8 to mediate the salt response [25]. In banana, MaVQ5 might act as a repressor of MaWRKY26 in the regulation of JA biosynthesis in response to cold stress [30]. In bamboo, PeVQ28 and WRKY83 interacted in the nucleus, and the overexpression of PeVQ28 in Arabidopsis increased resistance to salt stress and enhanced sensitivity to ABA [31]. In the present analysis, W-box elements, the binding sites of WRKY proteins, were identified in the promoters of 16 *CsVQ* genes, with an average frequency of 0.7 (Table. S1). The value was similar to that of *CmVQ*, and lower than that of the *AtVQ* and *VvVQ* [6, 15]. In Arabidopsis, apple, and soybean, VQ proteins only physically interact with WRKY transcription factors of Group I or Group IIc [6, 8, 14]. Twelve CsWRKYs of group I and group IIc, which were highly conserved with the corresponding Arabidopsis WRKY proteins, were presumed to interact with different CsVQ proteins (Fig. 5).

SA and MeJA are important endogenous signals mediating plant defenses [12, 48]. Previous studies have indicated that *VQ* genes are regulated by SA and MeJA treatments [6, 10, 17, 24], implying that they are likely involved in the responses to defense-related hormones. *VQ* genes in Arabidopsis [6], strawberry [12], and melon [16] were demonstrated to be responsive to SA and MeJA treatments to varying degrees. In *A. thaliana*, *AtVQ21*, *AtVQ22* can be inhibited by SA, but activated by JA [6, 25, 49], while *AtVQ23* can be induced by both SA and JA treatments [6, 34]. *CmVQ21–1*, *CmVQ22–1*, and *CmVQ23* in melon displayed similar trends with *AtVQ21*, *AtVQ22*, and *AtVQ23*, respectively. In the present study, the highly expressed *CsVQ* genes, including *CsVQ4*, *CsVQ6*, *CsVQ16–2, CsVQ19*, *CsVQ24*, and *CsVQ34*, were induced by MeJA and SA to varying degrees (Fig. 7), indicating that they might play crucial roles in SA- and JA-regulated defense. Almost all highly expressed *CsVQ* genes are involved in the response to hormones, indicating that *CsVQs* may participate in the regulation of cucumber abiotic stresses through different hormone-mediated signal transduction pathways.

Low temperature, salinity, and drought are common abiotic stresses that impair plant growth and development. Exogenous application of ABA to higher plants can accelerate their adaptation to these stresses by changing the expression of resistance genes [50], particularly for *CsVQ* genes (*CsVQ4*, *CsVQ6*, *CsVQ16–2*, *CsVQ19*, *CsVQ24*, *CsVQ30*, *CsVQ32*, *CsVQ33*, and *CsVQ34*) with higher expression levels in different tissues (Fig. 6). *CsVQ4* is homologous to *AtVQ4* (*MVQ1*) and *SlVQ6*, both of which are involved in the response to salt and drought stresses [11, 18]. In our analysis, *CsVQ4*, *CsVQ6*, *CsVQ16–2*, *CsVQ19*, and *CsVQ24* were significantly upregulated by low temperature, salinity, drought, and ABA treatment (Figs. 7 and 8). Similar results were obtained for ABA-treated *VQ* genes in rice, Moso bamboo, and melon [10, 16, 17]. *CsVQ16–2* exhibited drastically similar up-expression trends under abiotic stress and ABA treatments (Fig. 7). In cucumber, *CsVQ16–2* responded to salt, drought, and cold stresses (Fig. 8). In Arabidopsis, *AtVQ16* (*SIB2*), homologous to *CsVQ16–2* in cucumber, counteracts adversity by specifically identifying the C-terminal WRKY domain, which acts as a co-activator of AtWRKY33 in plant defense [34]. These results suggest that these *CsVQ* genes might participate in the ABA-dependent signaling transaction pathway to enhance plant adaptation to abiotic stresses. Further studies are required to identify presumed VQ interacting proteins and elucidate the signaling pathways in which they are involved.

## Conclusions

In conclusion, this study provides the comprehensive and systematic analysis of the *VQ* gene family in cucumber plants. A total of 32 VQ motif-containing proteins were identified and divided into 9 groups in cucumber. Genome-wide bioinformatics of *CsVQ* genes were performed to study the gene characteristics, evolution, and interaction networks. Furthermore, expression profiles of *CsVQ* genes were carried out to determine their potential functions in the growth, development and stress response in cucumber plant. *CsVQ* genes play an active part in the regulation of growth development, and response to salt, drought, and cold stress of cucumber plant, which may be closely related to the interactions of CsVQ proteins with CsWRKY transcription factors. These results will provide a basis for further research on biological functional differentiation, molecular mechanisms, and the role of *VQ* genes in abiotic stress response in cucumber.

## Methods

### Plant materials and stress treatments

Cucumber (*C. sativus* cv. Xintaimici, donated by the Prof. Xiaolei Sui from China Agricultural University) plants were grown in a phytotron for 16/ 8 h with day/night temperatures set at 25/ 18 °C. Cucumber plants at the 3–4 true-leaf stage were used for the stress and hormone treatments. For salt and drought stress, 250 mM sodium chloride and 20% (w/v) PEG 6000, instead of water, were used to irrigate cucumber seedlings respectively. For low-temperature treatment, seedlings were exposed to 6 °C for 48 h. For hormone treatment, leaves were sprayed with 100 μM ABA, 200 μM SA, and 175 μM MeJA [51]. The first and second true leaves of treated cucumber plants were randomly collected at 0, 1, 3, 6, 9, 12, and 24 h after treatment, then rapidly frozen in liquid nitrogen, and stored at − 80 °C until analysis.

### Sequence retrieval and identification, chromosomal location, gene structure, and promoter analysis of *CsVQ* genes

To identify the *VQ* genes in cucumber plants, VQ proteins from *A. thaliana* [6] were used as queries in cucumber ‘Chinese Long’ v2 genome (http://www.cucurbitgenomics.org/organism/2) in the CuGenDB (http://www.cucurbitgenomics.org/) [37, 52]. In addition, motif ID “PF05678” was used as the keyword for query in the cucumber database, and VQ motif-containing proteins were rechecked and confirmed by using the InterPro program (http://www.ebi.ac.uk/inter pro/) and SMART program (http://smart.embl.de/).

The length, molecular weight (MW), and theoretical isoelectric point (pI) of VQ proteins were calculated using the ProtParam tool (https://web.expasy.org/protparam/). The chromosomal locations of the *CsVQ* genes were determined using MapChart 2.3.2 [53]. Intron/exon structure analysis was performed using the Gene Structure Display Server (http://gsds.cbi.pku.edu.cn). CDS and genomic sequences of *CsVQ* genes were submitted to obtain the gene structure and draw diagram. Subcellular locations of CsVQ proteins were predicted using the Euk-mPLoc 2.0 server (http://www.csbio.sjtu.edu.cn/bioinf/euk-multi-2/) [54].

A sequence of 1500 bp upstream from the start codon of each *CsVQ* gene was downloaded from cucumber genome. Then *cis*-elements in promoter of each *CsVQ* gene were predicted by using the PlantCARE server (http://bioinformatics.psb.ugent.be/webtools/plantcare/html/) [55].

### Sequence alignment, conserved motif, and phylogenetic analysis

Amino acid sequences of the VQ proteins in cucumber were aligned by using DNAMAN version 9.0 (LynnonBiosoft, Quebec, Canada) [56]. Thirty-four Arabidopsis VQ protein sequences and 40 rice VQ protein sequences were downloaded from the TAIR database (https://www.arabidopsis.org/) and rice data sites (http://www.ricedata.cn/gene/), respectively. By using the maximum likelihood method [57] in MEGA X [58], the full-length sequences of VQ proteins from cucumber, Arabidopsis, rice, and tomato were compared to construct a phylogenetic tree. The distribution of conserved motifs of *CsVQ* in cucumber was analyzed using the MEME website (http://meme-suite.org/) with 10 maximum numbers of motifs.

### Analysis of interaction networks of CsVQ proteins

Functional interaction network models of CsVQ proteins were established using the STRING database (https://string-db.org), and the confidence parameters were set as 0.40 threshold. The interaction between CsVQs and CsWRKYs was predicted using PAIR website (http://www.cls.zju.edu.cn/pair/), and the interaction network was constructed using Cytoscape 3.7.2 [59].

### RNA extraction and cDNA reverse transcription

Total RNA was isolated from the leaves of cucumber plants using an RNA Extraction Kit (Huayueyang, China) according to the manufacturer’s instructions. Briefly, 50 ~ 100 mg of leaf tissues were quickly ground into powder in liquid nitrogen, transferred into Eppendorf tubes, and then immediately mixed by vortexing in lysis buffer. Prior to centrifuging at 12,000 rpm for 10 min at 4 °C, chloroform was added, blended by vortexing for 15 s, and incubated for 3 min at room temperature to phase separation. After transferring supernatant to new RNase-free tubes, potential RNA was precipitated, thoroughly rinsed by wash buffer, and then eluted by adding RNase-free water onto the membrane of spin column. Concentration and purity of total RNA were assessed in a NanoDrop 2000c Spectrophotometer (Thermo Scientific, USA), using a 1 μl aliquot of the total RNA solutions. RNA purity was estimated from the A260\A280 absorbance ratio. The integrity of total RNA was evaluated by running samples on 1.5% agarose gels. The ratio of the peak areas (28 S\18 S) corresponding to the 28 S and 18 S ribosomal RNAs was used as a reference for RNA degradation.

Reverse transcription was performed using a Hifair® II 1st Strand cDNA Synthesis Kit (gDNA digester plus, Yeasen, China) according to the manufacturer’s protocol. Total RNA samples were treated with RNase-free DNase and used for cDNA synthesis. The transcripts of *CsVQ* genes were analyzed by qRT-PCR using SYBR® *Premix Ex Taq*™ II (TakaRa, Japan) on a Bio-Rad IQ5 Real-Time PCR System (Bio-Rad, USA). Gene-specific primers (Supplement Table S2) were designed by using Primer Premier 6. The PCR conditions consisted of denaturation at 95 °C for 30 s, followed by 40 cycles of 95 °C for 5 s, and 60 °C for 34 s. For relative quantification, the cucumber *α-TUBULIN* gene was used as the internal control, and the relative expression levels were repeated in triplicate using the 2^–ΔΔCT^ method [60].

## Supplementary Information

**Additional file 1: Figure S1.** Intron and exon structures of the *VQ* genes in cucumber. The majority of the *CsVQ* genes only have one exon, except *CsVQ2, CsVQ4, CsVQ7*, *CsVQ9, CsVQ25,* and *CsVQ27*, which have two exons. **Figure S2.** *Cis*-elements in the promoters of *CsVQ* genes. **Supplementary Table S1.** Number of *cis*-elements related to various environmental stresses in the promoters of *CsVQ* genes. **Supplementary Table S2.** Primers used for quantitative PCR analysis in this study.

## Data Availability

Most data generated or analysed during this study are included in this article and its supplemental files. The sequencing data (bioproject PRJNA312872 of Cucurbit Expression Atlas) used and analyzed during this study is available in the Cucurbit Genomics Database.

## Abbreviations

ABA: Abscisic acid
CuGenDB: Cucurbit Genomics Database
JA: Jasmonic acid
MeJA: Methyl jasmonate
qRT-PCR: Quantitative real-time polymerase chain reaction
SA: Salicylic acid
VQ: Valine-glutamine
