# Supplementary material for: Genome-wide analysis of valine-glutamine motif-containing proteins related to abiotic stress response in cucumber (Cucumis sativus L.)
Source: BMC Plant Biol. 2021 Oct 25;21:492. doi: 10.1186/s12870-021-03242-9 (PMC8546950; doi:10.1186/s12870-021-03242-9)
Supplement: Supplementary file 1 — Additional file 1: Figure S1. Intron and exon structures of the VQ genes in cucumber. The majority of the CsVQ genes only have one exon, except CsVQ2, CsVQ4, CsVQ7, CsVQ9, CsVQ25, and CsVQ27, which have two exons. Figure S2. Cis-elements in the promoters of CsVQ genes. Supplementary Table S1. Number of cis-elements related to various environmental stresses in the promoters of CsVQ genes. Supplementary Table S2. Primers used for quantitative PCR analysis in this study. [file 12870_2021_3242_MOESM1_ESM.docx]

**
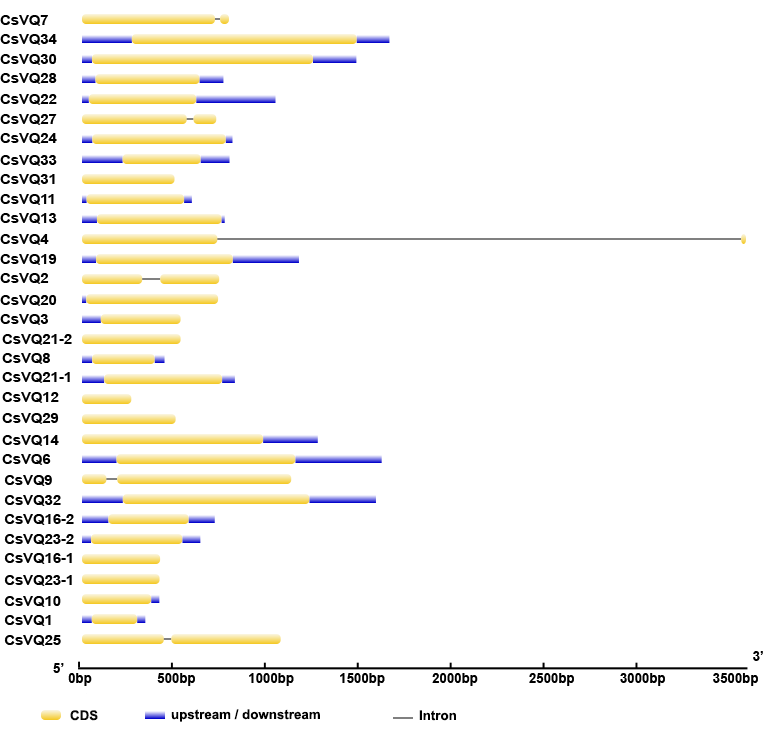
Fig. S1 Intron and exon structure of the *VQ* genes in cucumber.** The majority of the *CsVQ* genes only have one exon, except *CsVQ2, CsVQ4, CsVQ7*, *CsVQ9, CsVQ25, CsVQ27*, which have two exons.


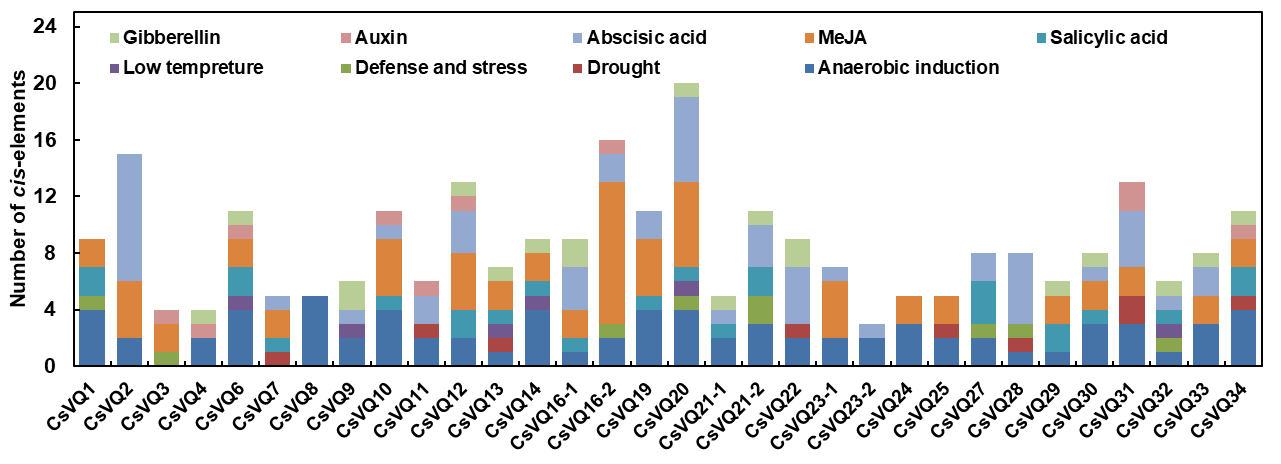


**Fig. S2 *Cis*-elements in the promoters of *CsVQ* genes.**

| **Supplementary Table S1. The number of *cis*-elements related to various environment stresses in promoter of *CsVQ* genes.** | | | | | | | | | | | | | |
| --- | --- | --- | --- | --- | --- | --- | --- | --- | --- | --- | --- | --- | --- |
|  | **Anaerobic induction** | **Drought** | **Defense and stress** | **Low temperature** | **Salicylic acid** | **MeJA** | **Abscisic acid** | **Auxin** | **Gibberellin** | **W box** | **Light** | **Endosperm expression** | **Meristem expression** |
| *CsVQ1* | 4 | 0 | 1 | 0 | 2 | 2 | 0 | 0 | 0 | 2 | 7 | 0 | 0 |
| *CsVQ2* | 2 | 0 | 0 | 0 | 0 | 4 | 9 | 0 | 0 | 0 | 24 | 0 | 0 |
| *CsVQ3* | 0 | 0 | 1 | 0 | 0 | 2 | 0 | 1 | 0 | 1 | 9 | 1 | 0 |
| *CsVQ4* | 2 | 0 | 0 | 0 | 0 | 0 | 0 | 1 | 1 | 1 | 12 | 0 | 0 |
| *CsVQ6* | 4 | 0 | 0 | 1 | 2 | 2 | 0 | 1 | 1 | 0 | 6 | 0 | 0 |
| *CsVQ7* | 0 | 1 | 0 | 0 | 1 | 2 | 1 | 0 | 0 | 2 | 9 | 0 | 0 |
| *CsVQ8* | 5 | 0 | 0 | 0 | 0 | 0 | 0 | 0 | 0 | 0 | 7 | 0 | 0 |
| *CsVQ9* | 2 | 0 | 0 | 1 | 0 | 0 | 1 | 0 | 2 | 3 | 4 | 0 | 0 |
| *CsVQ10* | 4 | 0 | 0 | 0 | 1 | 4 | 1 | 1 | 0 | 1 | 10 | 2 | 0 |
| *CsVQ11* | 2 | 1 | 0 | 0 | 0 | 0 | 2 | 1 | 0 | 0 | 3 | 1 | 0 |
| *CsVQ12* | 2 | 0 | 0 | 0 | 2 | 4 | 3 | 1 | 1 | 1 | 8 | 0 | 2 |
| *CsVQ13* | 1 | 1 | 0 | 1 | 1 | 2 | 0 | 0 | 1 | 0 | 11 | 0 | 0 |
| *CsVQ14* | 4 | 0 | 0 | 1 | 1 | 2 | 0 | 0 | 1 | 1 | 7 | 0 | 0 |
| *CsVQ16-1* | 1 | 0 | 0 | 0 | 1 | 2 | 3 | 0 | 2 | 0 | 15 | 0 | 0 |
| *CsVQ16-2* | 2 | 0 | 1 | 0 | 0 | 10 | 2 | 1 | 0 | 1 | 11 | 0 | 0 |
| *CsVQ19* | 4 | 0 | 0 | 0 | 1 | 4 | 2 | 0 | 0 | 0 | 8 | 1 | 1 |
| *CsVQ20* | 4 | 0 | 1 | 1 | 1 | 6 | 6 | 0 | 1 | 0 | 17 | 0 | 0 |
| *CsVQ21-1* | 2 | 0 | 0 | 0 | 1 | 0 | 1 | 0 | 1 | 1 | 11 | 0 | 0 |
| *CsVQ21-2* | 3 | 0 | 2 | 0 | 2 | 0 | 3 | 0 | 1 | 0 | 11 | 0 | 0 |
| *CsVQ22* | 2 | 1 | 0 | 0 | 0 | 0 | 4 | 0 | 2 | 0 | 21 | 0 | 1 |
| *CsVQ23-1* | 2 | 0 | 0 | 0 | 0 | 4 | 1 | 0 | 0 | 1 | 8 | 0 | 0 |
| *CsVQ23-2* | 2 | 0 | 0 | 0 | 0 | 0 | 1 | 0 | 0 | 2 | 8 | 0 | 0 |
| *CsVQ24* | 3 | 0 | 0 | 0 | 0 | 2 | 0 | 0 | 0 | 0 | 14 | 0 | 0 |
| *(Continued)* |  |  |  |  |  |  |  |  |  |  |  |  |  |
| *CsVQ25* | 2 | 1 | 0 | 0 | 0 | 2 | 0 | 0 | 0 | 0 | 8 | 0 | 0 |
| *CsVQ27* | 2 | 0 | 1 | 0 | 3 | 0 | 2 | 0 | 0 | 1 | 12 | 0 | 0 |
| *CsVQ28* | 1 | 1 | 1 | 0 | 0 | 0 | 5 | 0 | 0 | 1 | 15 | 0 | 0 |
| *CsVQ29* | 1 | 0 | 0 | 0 | 2 | 2 | 0 | 0 | 1 | 0 | 5 | 0 | 0 |
| *CsVQ30* | 3 | 0 | 0 | 0 | 1 | 2 | 1 | 0 | 1 | 0 | 11 | 1 | 0 |
| *CsVQ31* | 3 | 2 | 0 | 0 | 0 | 2 | 4 | 2 | 0 | 3 | 7 | 0 | 0 |
| *CsVQ32* | 1 | 0 | 1 | 1 | 1 | 0 | 1 | 0 | 1 | 0 | 9 | 0 | 0 |
| *CsVQ33* | 3 | 0 | 0 | 0 | 0 | 2 | 2 | 0 | 1 | 0 | 6 | 2 | 1 |
| *CsVQ34* | 4 | 1 | 0 | 0 | 2 | 2 | 0 | 1 | 1 | 1 | 6 | 0 | 0 |

| **Supplementary Table S2. Primers used for quantitative PCR analysis in this study.** | | |
| --- | --- | --- |
| **Description** | **Direction** | **Sequence (5’ - 3’)** |
| *CsVQ4* | F | CTGGGTTGGCGAACTGTAGC |
|  | R | AGTCCCGAGGAGTGGTGGTC |
| *CsVQ6* | F | CCGACTTCCCAGTTTCTATT |
|  | R | GGACCTAAGATCCCTGATTG |
| *CsVQ16-2* | F | CCTGACCCGACTAAGTTCTA |
|  | R | GTTCTTGCTCATCCTCCTCT |
| *CsVQ19* | F | CGTTGAATGATGACCCGTTGT |
|  | R | ATCGGCGATGGATGCAAGTA |
| *CsVQ24* | F | GATGGACTTCCGACGCTTGA |
|  | R | GACCCGATTGGACCGTGATT |
| *CsVQ30* | F | ATGGATTCTGGGAGTAGTGG |
|  | R | ATTAGCCTTAGGACCGACAT |
| *CsVQ32* | F | GCCACAGCAGCAACAACAACC |
|  | R | TAACGGAGGCAGAGGCGAAAG |
| *CsVQ33* | F | GCTGACCCATCAACATTCCG |
|  | R | GCGACACCATCCCGTTTCCA |
| *CsVQ34* | F | AACCGAGTTTATTTATTGCC |
|  | R | CATACTTCCACTATTCCCAGA |
